# Study on the key parameters of ice particle air jet ejector structure

**DOI:** 10.1038/s41598-024-68869-8

**Published:** 2024-08-01

**Authors:** Wang Man, Niu Zehua, Yong Liu

**Affiliations:** 1 State Key Laboratory of Coking Coal Resources Green Exploitation, Pingdingshan, 467000 China; 2 State Key Laboratory Cultivation Base for Gas Geology and Gas Control, Henan Polytechnic University, 2001 Shiji Road, Jiaozuo, 454000 Henan China

**Keywords:** Surface treatment, Paint removal technology, Ice particle jet, Jet pump, Nozzle design, Engineering, Mechanical engineering, Coarse-grained models

## Abstract

Existing ice particle jet surface treatment technology is prone to ice particle adhesion during application, significantly affecting surface treatment efficiency. Based on the basic structure of the jet pump, the ice particle air jet surface treatment technology is proposed for the instant preparation and utilization of ice particles, solving the problem of ice particle adhesion and clogging. To achieve efficient utilization of ice particles and high-speed jetting, an integrated jet structure for ice particle ejection and acceleration was developed. The influence of the working nozzle position (*L*_d_), expansion ratio (*n*), and acceleration nozzle diameter ratio (*D*_n_) length-to-diameter ratio (*L*_n_) on the ice particle ejection and acceleration was systematically studied. The structural parameters of the ejector were determined using the impact kinetic energy of ice particles as the comprehensive evaluation index, and the surface treatment test was conducted to verify the results. The study shows that under 2 MPa air pressure, the ejector nozzle parameters of *n* = 1.5, *D*_n_ = 4.0, *L*_d_ = 4, and *L*_n_ = 0 mm can effectively eject and accelerate the ice particles. The aluminum alloy plate depainting test obtained a larger paint removal radius and resulted in a smoother aluminum alloy plate surface, reducing the surface roughness from 3.194 ± 0.489 μm to 1.156 ± 0.136 μm. The immediate preparation and utilization of ice particles solved the problems of adhesion and storage in the engineering application of ice particle air jet technology, providing a feasible technical method in the field of material surface treatment.

## Introduction

Material surface treatment plays a crucial role in anticorrosion and prolonging the service life of materials^1–3^. Traditional methods, such as sandblasting, electroplating brush treatment, and chemical treatment^4,5^. are limited by environmental pollution, high energy consumption, and health hazards^6–10^, which constrain their widespread adoption.

Consequently, the development of clean and environmentally friendly surface treatment technologies is essential. ARASH et al.^11^ investigated atmospheric pressure plasma metal surface treatment technology, which employs microwave-assisted atmospheric plasma for the quantitative removal of pollutants, thereby avoiding environmental pollution and achieving high cleaning speed. However, this technology’s high environmental requirements hinder its broader application. Nuo Jin et al.^12^ proposed micro-nanometer bubble surface treatment technology, which is environmentally friendly and minimally damaging to substrate materials. Nevertheless, its industrial application is limited by the high precision required for equipment and low processing efficiency. In response to the need for reducing energy consumption in green cleaning technologies and improving engineering applicability, researchers have proposed ice particle jet surface treatment technology. This method, which uses air jets of ice particles, offers numerous advantages, most notably that the ice abrasive melts into water and is immediately discharged after operation, eliminating the need for abrasive recycling and dust generation^13^. The first ice abrasive jet technology patent involved using ice abrasives for surface formation, akin to sandblasting and paint removal^14^. This method utilizes the strength and hardness of ice particles to create a high-speed abrasive stream driven by a high-speed fluid to clean, strip paint, and remove rust.

The ice-making method is crucial for this technology. Initially, the ice-breaking method was used to prepare ice particles, which were then accelerated by jets of different media for surface treatment. For instance, Liu et al.^15^ crushed ice and used high-speed air negative pressure to suction and accelerate ice particles, effectively removing surface paint from workpieces. Geskin et al.^16^ from the New Jersey Institute of Technology prepared ice particles using the ice-breaking method, transported them to the nozzle with low-temperature airflow, and mixed them with a high-speed water stream to form an ice-particle water jet for cutting metals and soft materials. However, this method produces varying ice particle sizes and struggles to create particles smaller than millimeters.

To overcome the limitations of the ice-breaking method, Shin. et al.^17^ used liquid droplets in a vacuum environment to continuously produce spherical ice particles with a diameter of 50 μm and a temperature of 0 °C. However, this method is equipment-intensive, complex, has high energy consumption, and low production efficiency, and cannot control the ice particle size and temperature. Li Deyu^18^ prepared ice pellets by cooling liquid droplets with liquid nitrogen spray, achieving a high preparation efficiency and an average particle size of 100 μm. However, these ice particles easily bonded at temperatures above − 40 °C, blocking the ice particle outlet^19^.

Despite the advantages of ice particle jet technology in surface treatment, it has not seen widespread engineering application due to unresolved issues in ice particle preparation and storage technology. To address these challenges, the authors proposed a technology for preparing ice particles with controllable size and hardness, based on instant preparation and utilization^20^. They used a jet pump ejector to instantly eject and accelerate ice particles, integrating preparation and utilization, thus solving issues of uncontrollable particle size and avoiding adhesion and high energy consumption during storage. Efficient ejector performance is key to the immediate utilization of ice particles and effective jet surface treatment.

Ejector technology is widely used for abrasive jets, effectively mixing fluid–solid mixtures^21,22^. Bohnet et al.^23^ conducted conveying tests with various powder materials, analyzing the variation of gas velocity, solid particle velocity, and hydrostatic pressure along the axial direction for a given feed. They found that the nozzle location significantly affects the ejector’s operating characteristics. Chellappan^24^ studied a moment-type ejector using wheat as the experimental material and found that the ejector’s performance is influenced by nozzle positioning. Similarly, Dawson^25^ and Davies et al.^26^ conducted studies on parameters affecting ejector performance, with their conclusions aligning with existing literature.

Xiong et al.^27^ used simulation to obtain the static pressure distribution inside the ejector, discovering that the working nozzle’s position impacts the static pressure magnitude, which in turn affects the abrasive’s priming rate. Feng et al.^28^ designed a liquid nitrogen-ice particle jet working nozzle structure based on jet pump technology and verified its ability to eject ice particles timely and achieve good processing results through testing. Zhang et al.^29^ examined the impact of the working nozzle dispersal section length on ejector performance, concluding that a well-designed dispersal section length can significantly enhance processing efficiency. Overall, existing research focuses on the ejection capacity of the ejector but lacks studies on the fluid–solid acceleration performance.

Therefore, this paper examines the impact of injector structural parameters on the ejection and acceleration of ice particles, aiming to improve surface treatment efficiency based on the principle of instant preparation and utilization of ice particles in air jet technology. The injector mainly comprises working nozzles, a mixing chamber, and accelerating nozzles. Instantly prepared ice particles are sucked into the mixing chamber by gravity and the high-speed airflow’s negative pressure from the working nozzle. The high-speed airflow then introduces the ice particles into the accelerating nozzle for thorough mixing and acceleration before ejection. Previous studies^30–34^show that the working nozzle’s ejection capacity depends on the nozzle’s expansion ratio and position, while the accelerating nozzle’s acceleration capacity depends on its diameter ratio and aspect ratio. This study analyzes the ejection and acceleration of ice particles under different ejector nozzle structures using the ANSYS-FLUENT gas–solid two-phase flow model. A paint stripping test is conducted on the designed ejector to verify its surface treatment capability, providing theoretical support for the application of ice particle air jets in material surface treatment.

## Principle of ice air jet

**Figure 1:**
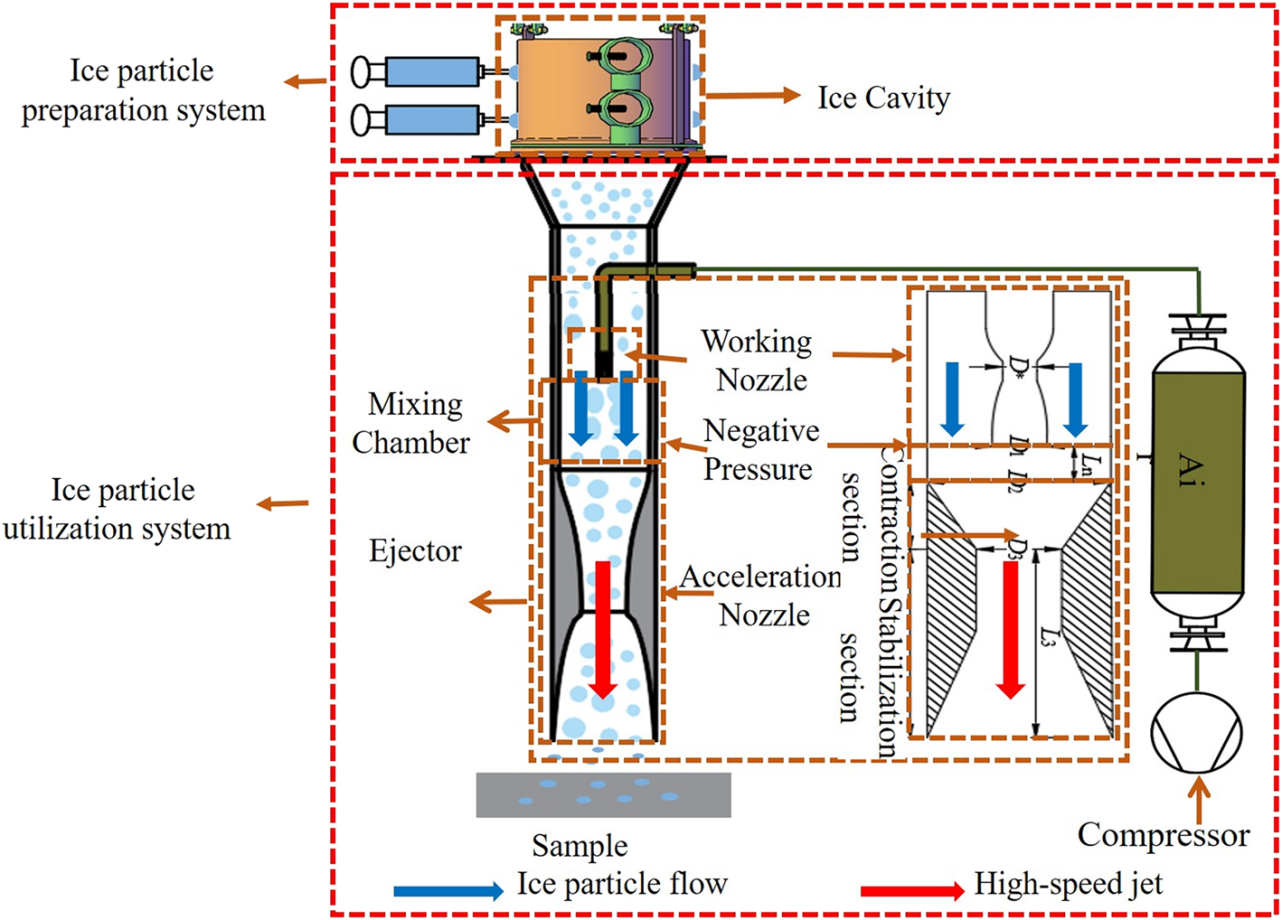
Principle of ice particle air jet injection.

The principle of ice particle air jet technology, based on the instant preparation and utilization of ice particles, is illustrated in Fig. 1. Using the principle of heterogeneous nucleation, water is injected into the syringe after being pressurized by the pump. Ice particle size is controlled by changing the needle model and adjusting the injection pressure, resulting in particles ranging from 0.1 mm to 2.52 mm. The temperature of the cold wall is regulated by adjusting the amount of liquid nitrogen added, which controls both the preparation efficiency and the temperature of the ice particles. Once the ice particles condense on the cold wall, a scraper moves from top to bottom to remove them, then resets. The speed of the scraper’s reciprocating movement and the number of needles determine the mass flow rate of ice particle preparation^17,18^.

In the ice pellet utilization system, 2 MPa of gas is passed through the ejector working nozzle to form a high-speed jet, creating a negative pressure area at the nozzle outlet. Instantly prepared ice particles are transported to this negative pressure area by low-pressure gas, ejected into the mixing chamber, mixed with the high-speed air jet, and initially accelerated. They then enter the accelerating nozzle for full acceleration before being ejected to form a high-speed ice particle air jet. The working efficiency of the ice particle air jet is determined by the kinetic energy of the impacting ice particles, which is a function of the mass and acceleration of the ejected ice particles. Therefore, the ejection and acceleration capabilities of the ejector are crucial for determining the efficiency of surface treatment.

## Numerical simulation

### Physical modeling and meshing

**Figure 2:**
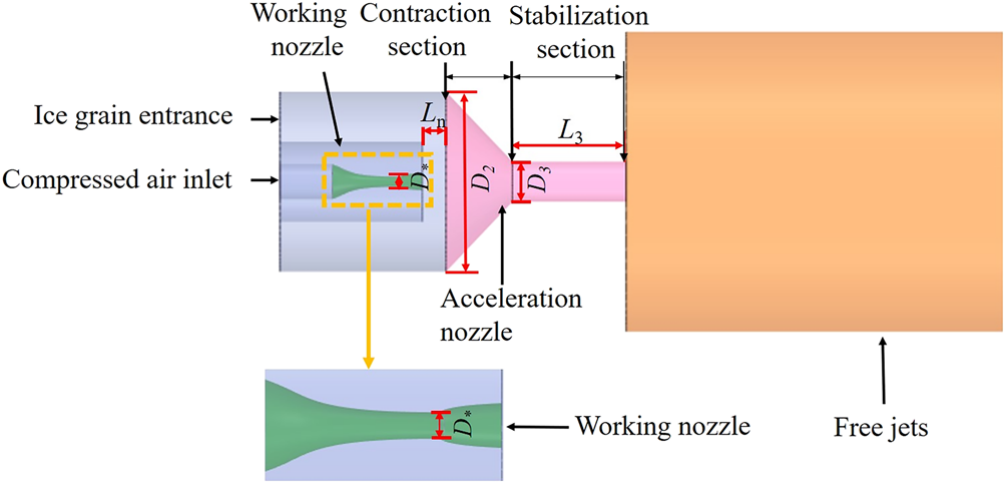
Physical modeling of ice particle air jets.

The ejection capacity of the injector is determined by the position of the working nozzle (*L*_n_) and the expansion ratio (*n*), which is the ratio of the static pressure at the working nozzle outlet cross-section to the ambient pressure. The acceleration capacity is determined by the diameter ratio (*D*_n_=*D*_3_/D*) and the length-to-diameter ratio (*L*_d_=*L*_3_/*D*_3_) of the accelerating nozzle, as shown in Fig. 2.

**Figure 3:**
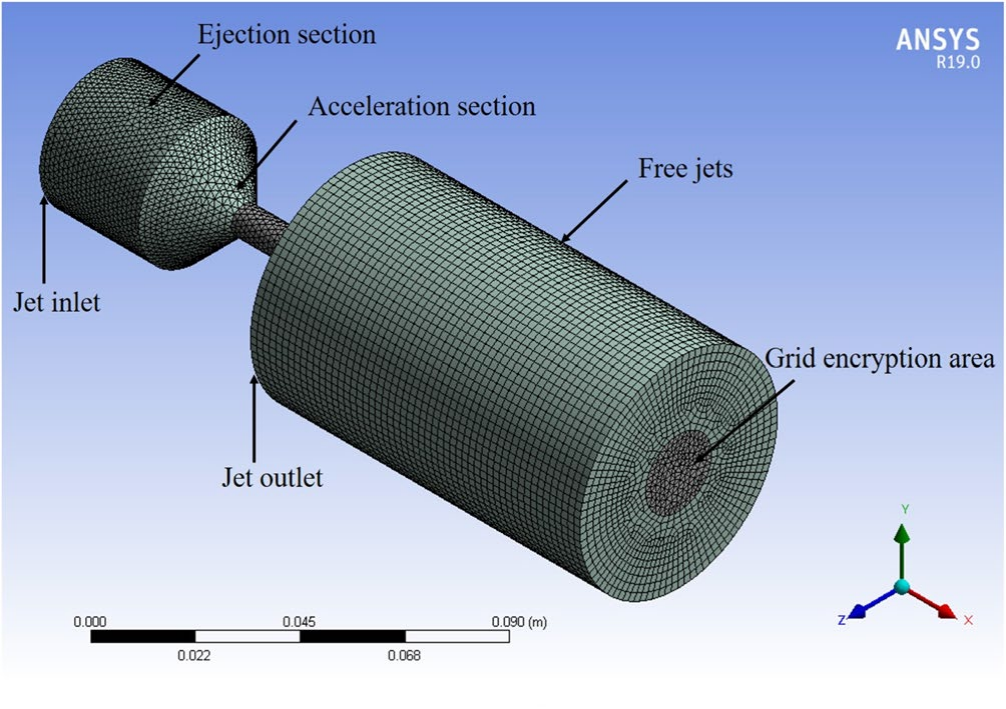
Grid division.

The meshing of the model is illustrated in Fig. 3. Using unstructured grids, quadrilateral elements were chosen as the dominant method to generate the computational domain. To improve computational accuracy, the working nozzle, accelerating nozzle, and the flow field and wall zones were regionally refined and validated for mesh independence^35^. According to research needs, the outer basin’s length along the flow direction is 100 mm, and its radius is 30 mm. This was accomplished using the ANSYS Mesh module in Workbench, resulting in approximately 176,000 meshes, with the minimum mesh volume of 6.29 × 10^−11^ m^3^, and an average mesh mass of 0.85.

### Numerical simulation of control equations

#### Particle phase control equation

In the Fluent-EDEM coupled calculations, the motion of a single ice particle follows Newton’s second law. The law of motion for an arbitrary ice particle *i* is expressed as follows^36^:

1 $$F_{i} = m_{i} \ddot{v}_{i}$$

2 $$M_{i} = I_{i} \ddot{\theta }_{i}$$

where $\ddot{v}_{i}$ and $\ddot{\theta }_{i}$ are the gravitational and angular acceleration of ice particle *i*, respectively, *m*_i_ and *I*_*i*_ are the mass and moment of inertia of particle *i*, respectively, and *F*_*i*_ and *M*_*i*_ are the combined force and torque, respectively.

Inside the nozzle and in the jet flow field, solid particles are subjected to various forces, some of which are small and can be neglected, such as virtual mass forces. This study considers trailing forces, particle interaction forces, pressure gradient forces, Magnus forces, and others. The forces on the particles are expressed as^36^:

3 $$F_{i} = m_{i} g + f_{{{\text{p}},i}} + \sum\nolimits_{i = 1}^{{k_{i} }} {(f_{{{\text{c}},ij}} + f_{{{\text{d}},ji}} )} + f_{{{\text{mag}},i}} + f_{{{\text{saff}},i}}$$

where *f*_p_,_*i*_ is the trailing force of particle* i*, *f*_c_,_*ij*_ is the collision force of particle *i* and particle *j*,* f*_d_,_*ij*_ is the damping force of particle* i* and *j*, fmag*,i* is the Magnus force of particle *i*, and fsaff,*i* is the Saffman lift force of particle *i*.

#### Heat transfer calculation equation

The ice particle air jets are calculated using the Hertz-Mindlin with Heat Conduction contact model, with the governing equations as follows ^37^:

① Hertz-mindlin(No Slip)
  The normal force *F*_n_ is expressed as:
  4 $$F_{{\text{n}}} = \frac{4}{3}E^{*} \sqrt {R^{*} } \delta_{{\text{n}}}^{\frac{3}{2}}$$
  where $E^{*}$ is the Young’s modulus, $R^{*}$ is radius of the contact sphere, *δ*_n_ is normal overlap function, respectively.
  w Rolling friction is considered by applying a moment to the contact surface:
  5 $$\tau_{i} = - \mu_{r} F{\text{n}}R_{i} \omega_{i}$$
  where *μ*_r_ is the coefficient of rolling friction, *R*_i_ is the distance from the contact point to the center of mass, and *ω*_*i*_ is the unit angular velocity vector of the object at the contact point.
② Hertz-mindlin with heat conduction
  In this study, the significant contact between particles necessitates considering heat transfer. The heat flow between particles is defined by:
  6 $$Q_{{{\text{p}}1{\text{p}}2}} = h{\text{c}}\Delta T_{{{\text{p}}1{\text{p}}2}}$$
  where the contact area is added to the heat transfer coefficient *h*_c_ , expressed as:
  7 $$h_{{\text{c}}} = \frac{{4k_{{{\text{p}}1}} k_{{{\text{p}}2}} }}{{k_{{{\text{p}}1}} + k_{{{\text{p}}2}} }}\left[ {\frac{{3F_{{\text{N}}} r^{*} }}{{4E^{*} }}} \right]^{1/3}$$
  where *F*_N_ is the normal force, r* is the geometrically averaged radius of the particles calculated from the Hertz elastic contact theory, and E* is the effective Young’s modulus.
③ Temperature Update Model
  The heat transfer algorithm is activated once the heat fluxes are calculated. The temperature change over time for each particle is updated by:
  8 $$m_{{\text{p}}} C{\text{p}}\frac{dT}{{dt}} = \sum {Q_{{{\text{heat}}}} }$$
  where *m*_p_, *C*_p_ and T are mass, specific heat capacity and temperature, respectively. The right-hand side represents the sum of convective and conductive heat fluxes.

### Initial and boundary conditions

The initial and boundary conditions set in EDEM and FLUENT software are shown in Table 1. Ice particles start moving from the injector inlet, with the initial kinetic energy of the ice particles being zero.

**Table 1:** Initial and boundary conditions.

Parameter	Value
Particle temperature/(K)	213.15
Particle density/(g cm^−3^)	0.9
Particle mass flow/(kg s^−1^)	0.01
Particle diameter/(m)	0.001
Crash recovery factor	0.9
Inlet pressure/(MPa)	2
Ambient temperature/(K)	300.15
Ambient pressure/(Pa)	101,325
Gas viscosity/(Pa s^-1^)	17.894 × 10^−6^
Gas specific heat/[J (kg∙K)^−1^]	1006
Gas thermal conductivity/[W (m∙K)^−1^]	0.0242

### Numerical simulation scheme

The numerical simulation considers the influence of the working and accelerating nozzles on the acceleration effect of ice particles. The test scheme is shown in Table 2.

**Table 2:** Numerical simulation scheme.

Number	*L*_n_/mm	*n*	*D*_n_	*L*_d_
1	− 5	1.5	4.5	4.0
2	0	1.0	4.0	5.0
3	5	0.8	3.5	3.0
4	− 5	0.8	3.5	3.0
5	0	1.0	4.5	4.0
6	5	1.5	4.0	5.0
7	− 5	0.8	3.5	3.0
8	0	1.5	4.0	4.0
9	5	1.0	4.5	5.0
10	− 5	0.8	3.5	3.0
11	0	1.5	4.0	4.0
12	5	1.0	4.5	5.0

**Figure 4:**
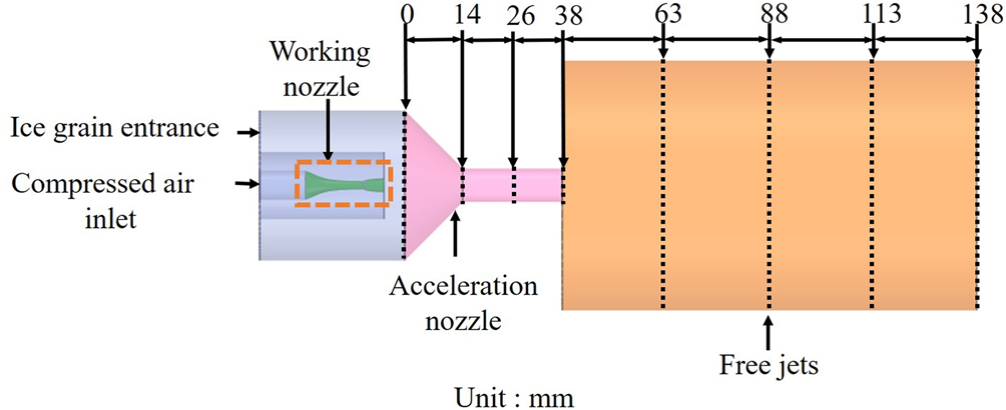
Schematic diagram of ice particle capture area of ejector.

To better investigate the acceleration law and process of the ice particle jet, an ice particle capture domain was established on the injector. The width of the capture domain was set to 1.5 times the particle diameter. The optimal structural parameters of the injector nozzle were determined by analyzing the gas velocity, negative gas pressure, and kinetic energy data of each capture domain. The location of the capture domain is illustrated in Fig. 4.

### Numerical simulation results and analysis

#### Effect of L_n_、n on the kinetic energy of ice particle impacts

##### Influence on the amount of ice particles elicited

**Figure 5:**
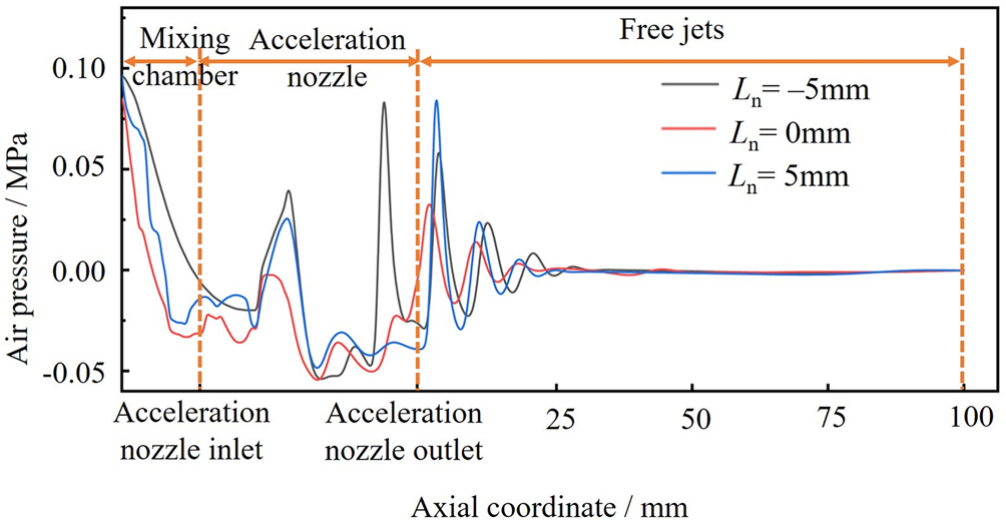
Effection of different Ln on the negative pressure of the mixing chamber.

Taking *n* = 1.5, *D*_n_ = 4.5, *L*_d_ = 4.0 as an example to illustrate the influence of the position of the working nozzle’s position on the gas’s negative pressure, the calculation results are shown in Fig. 5. It can be seen that different *L*_n_ injectors exhibit similar pressure distribution patterns. The gas pressure at the exit of the working nozzle drops significantly, reaching maximum negative pressure in the acceleration nozzle. After the acceleration nozzle, the gas pressure fluctuates and gradually converges to atmospheric pressure. However, *L*_n_ has a greater impact on the pressure in the mixing chamber. The different positions of the working nozzle affect the spatial pattern in the mixing chamber, which significantly influences the negative gas pressure. As *L*_n_ increases, the negative gas pressure inside the mixing chamber decreases, then increases, reaching its lowest at *L*_n_ = 0 mm. Therefore, it can be concluded that *L*_n_ = 0 mm is more favorable for the ejection of ice particles.

**Figure 6:**
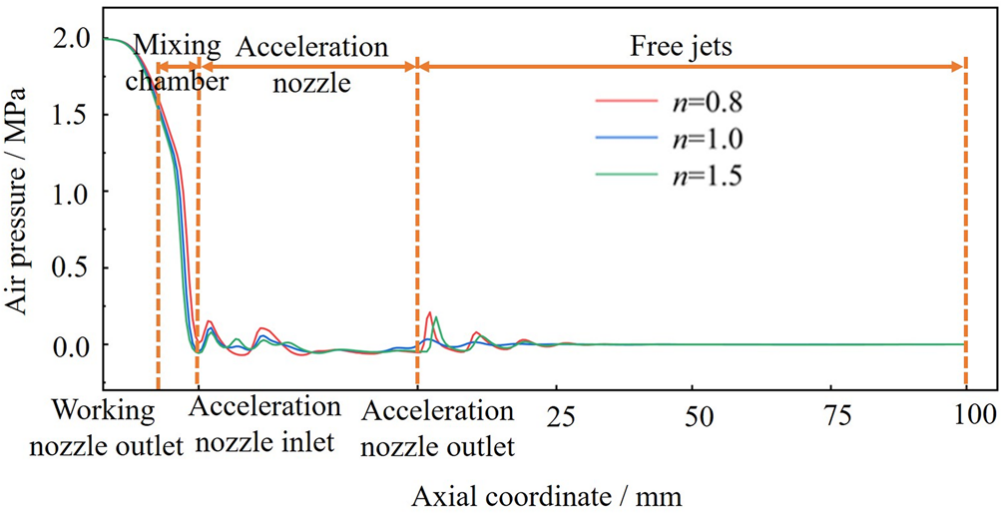
Effect of different n on the negative pressure of mixing chamber.

Taking* L*_n_ = 0 mm, *D*_n_ = 4.5, *L*_d_ = 4.0 as examples to illustrate the influence of the working nozzle expansion ratio on the negative pressure of the gas, the calculation results are shown in Fig. 6. It can be seen that the pressure distribution patterns formed by different working nozzle expansion ratios are basically the same. The gas pressure decreases significantly at the exit of the working nozzle, reaching a minimum at the entrance of the acceleration nozzle. The gas pressure then fluctuates within the acceleration nozzle and gradually stabilizes in the free jet section as the jet develops. Different working nozzle expansion ratios affect the jet morphology in the mixing chamber, which significantly impacts the negative gas pressure. As *n* increases, the gas pressure gradually decreases, and the negative pressure generated in the mixing chamber is the highest at *n* = 1.5. Therefore, it can be assumed that *n* = 1.5 is more favorable for the ejection of ice particles.

##### Effects on the initial acceleration of ice particles

**Figure 7:**
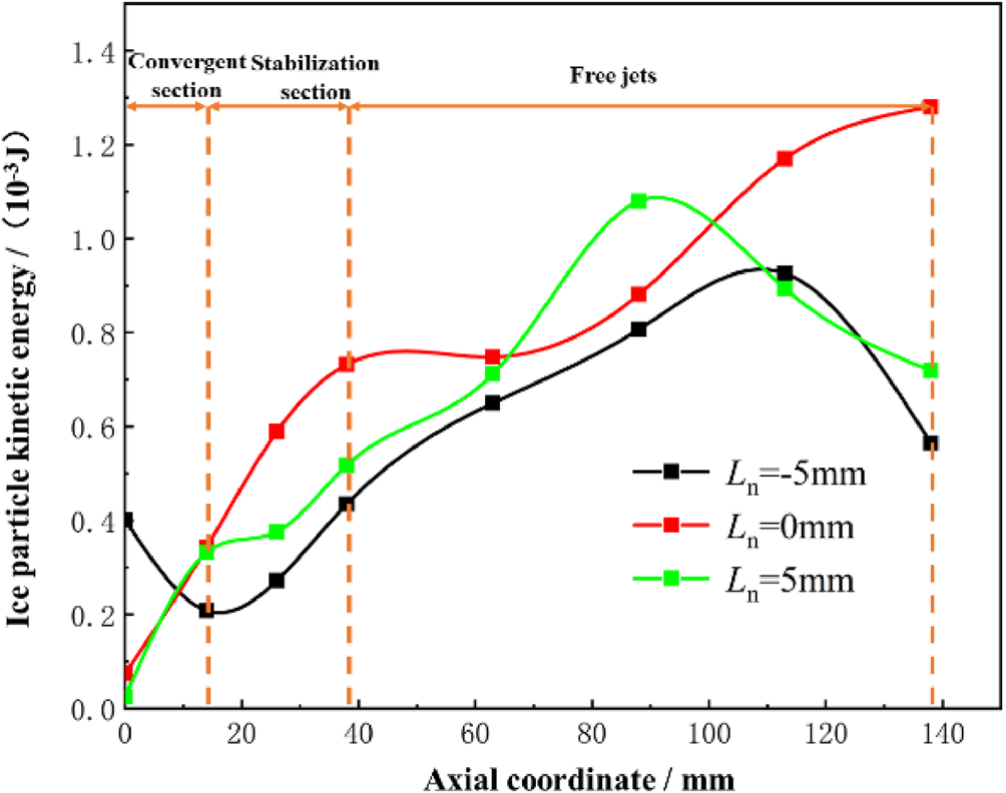
Variation of kinetic energy of ice particles at different Ln.

**Figure 8:**
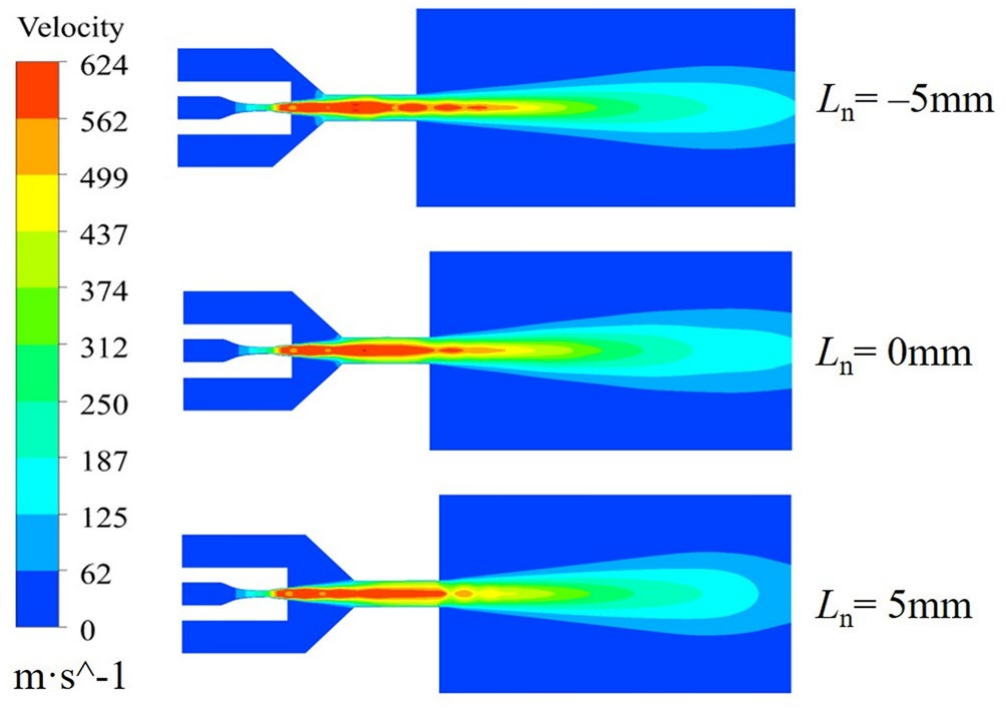
Airflow velocity clouds at different Ln.

Ice particles are initially accelerated by the high-speed airflow after elicitation, and the acceleration effect is characterized by the kinetic energy possessed by the ice particles. The effect of the working nozzle position on the impact kinetic energy of ice particles is illustrated with *n* = 1.5, *D*_n_ = 4.5, *L*_d_ = 4.0 as examples, and the calculated results are shown in Fig. 7. The kinetic energy of ice particles continues to increase after entering the accelerating nozzle, but decreases in the second half of the jet at *L*_n_ = − 5 mm and *L*_n_ = 5 mm. The kinetic energy of ice particles increases consistently at *L*_n_ = 0 mm reaching a maximum of 1.28 × 10^−3^ J**.** The different positions of the working nozzles affect the spatial morphology in the mixing chamber, which greatly impacts the acceleration of ice particles. As shown in the airflow velocity cloud plots at different *L*_n_ in Fig. 8, he airflow fluctuation is minimal at *L*_n_ = 0 mm, and the gas expands completely inside the accelerating nozzle, resulting in the best acceleration effect. In contrast, at *L*_n_ = -5 mm and* L*_n_ = 5 mm, the gas flow enters the free flow field with alternating expansion and compression waves, causing intense energy exchange with the surrounding environment, which reduces the jet flow velocity. Therefore, it can be concluded that *L*_n_ = 0 mm is more favorable for the initial acceleration of ice particles.

**Figure 9:**
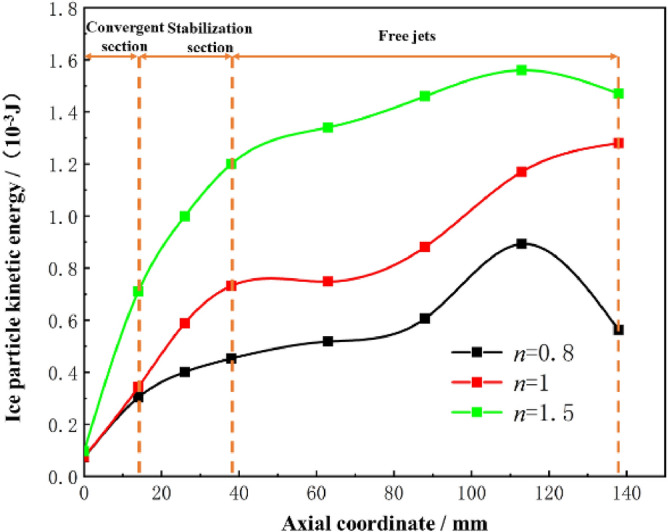
Variation of kinetic energy of ice particles with different n.

**Figure 10:**
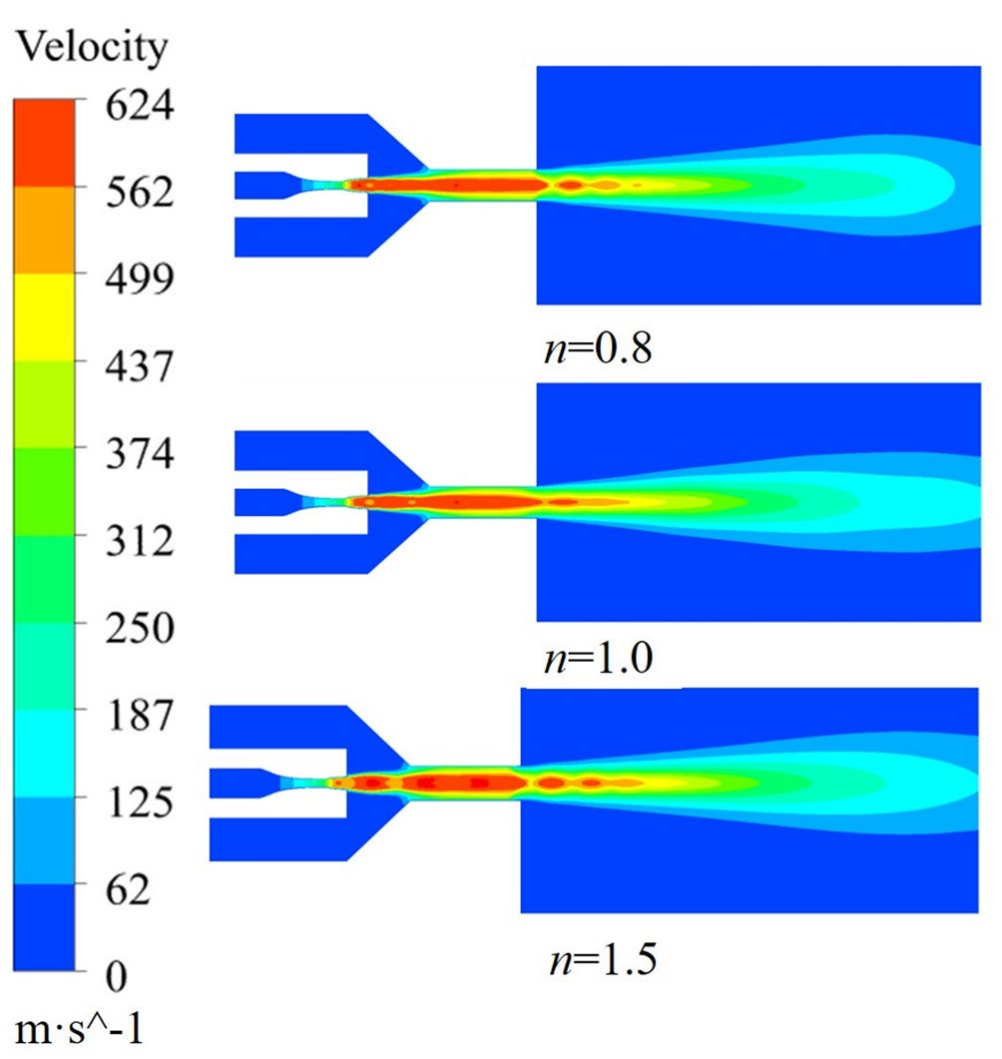
Airflow velocity clouds at different n.

The effect of the working nozzle expansion ratio on the acceleration of ice particles is illustrated with *L*_n_ = 0 mm, *D*_n_ = 4.5, *L*_d_ = 4.0 as examples. The calculation results are shown in Fig. 9. The kinetic energy of ice particles under different *n* values continues to increase after entering the accelerating nozzle. At the end of the jet flow field, the velocity of ice particles decreases at *n* = 0.8 and *n* = 1.5, but the kinetic energy of ice particles is always the highest at *n* = 1.5, reaching a peak value of 1.56 × 10^−3^ J at a target distance of 75 mm. Combined with the air velocity cloud diagrams under different *n* values in Fig. 10, it can be seen that the jet iso-velocity core is the longest and the jet velocity is the highest with a nozzle expansion ratio of *n* = 1.5, Although some fluctuations are generated in the free jet stage, the overall jet energy is higher, effectively accelerating the ice particles. The working nozzles with *n* = 0.8 and *n* = 1 have a limited acceleration effect on ice particles due to their lower jet velocities. Therefore, the nozzle with *n* = 1.5 provides the best initial acceleration effect on ice particles.

#### Effect of Dn、Ld on the kinetic energy of ice particle impacts

##### Effects on the gas velocity field

**Figure 11:**
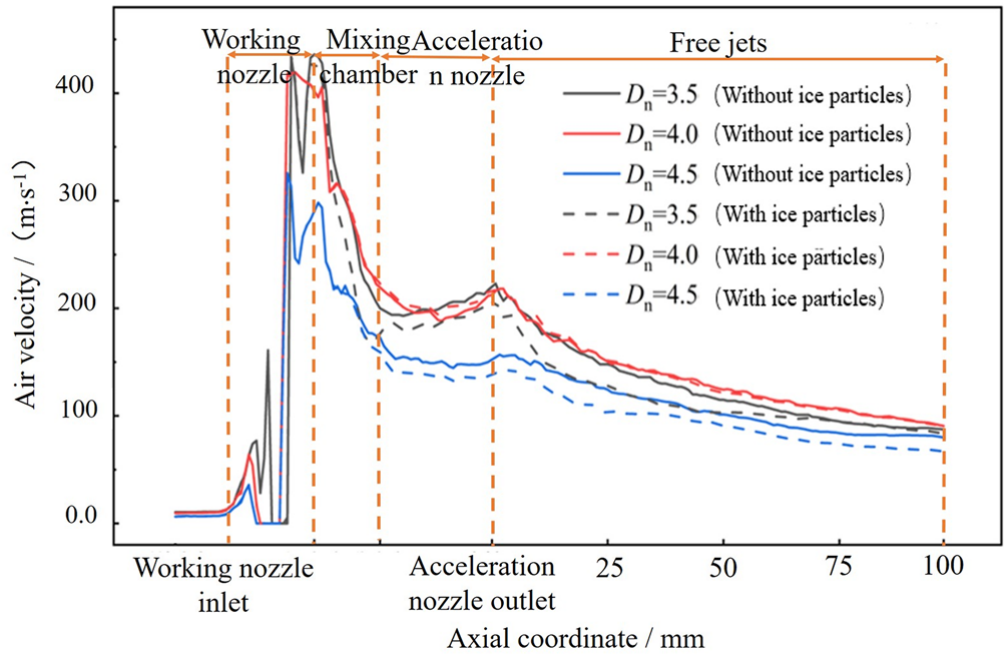
Axial velocity distribution of acceleration nozzle at different Dn.

**Figure 12:**
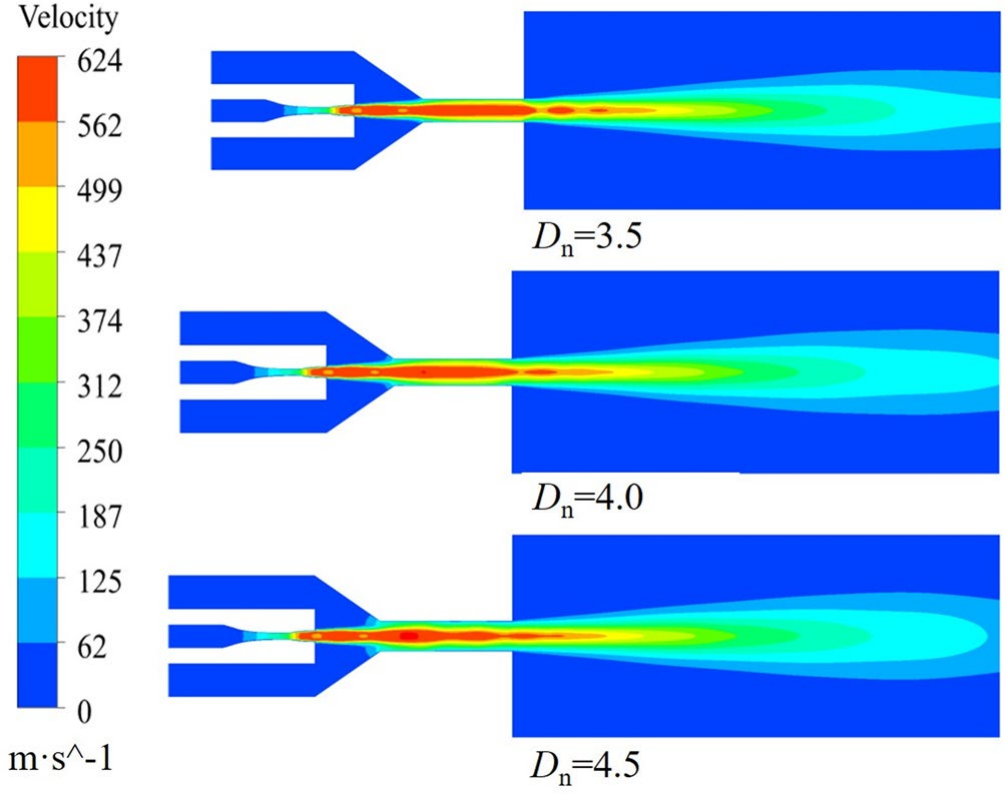
Airflow velocity clouds at different Dn.

The effect of *D*_n_ on the gas velocity is illustrated with *L*_n_ = 0 mm, *n* = 1.5 and *L*_d_ = 4.0 The calculation results are shown in Fig. 11. After entering the working nozzle, the gas velocity accelerates, but it starts to decrease gradually when entering the mixing chamber. It accelerates again after entering the accelerating nozzle, although the increase in velocity is not significant. In the free jet section, the gas velocity continues to decrease as the jet develops. Inside the accelerating nozzle, *D*_n_ significantly affects the gas velocity, which initially increases and then decreases as *D*_n_. increases. There is little difference between the gas velocities at *D*_n_ = 3.5 and *D*_n_ = 4.0, but the gas velocity at *D*_n_ = 4.5 is always the smallest. As shown in the gas velocity cloud diagram under different *D*_n_ values in Fig. 12, when *D*_n_ = 4.0, the gas flow fluctuation is minimal, resulting in lower energy loss. However, when *D*_n_ = 3.5, the gas fluctuation is large, and the energy exchange with the environment is intense, reducing the jet’s energy. Therefore, *D*_n_ = 4.0 is more conducive to stabilizing the gas flow field.

**Figure 13:**
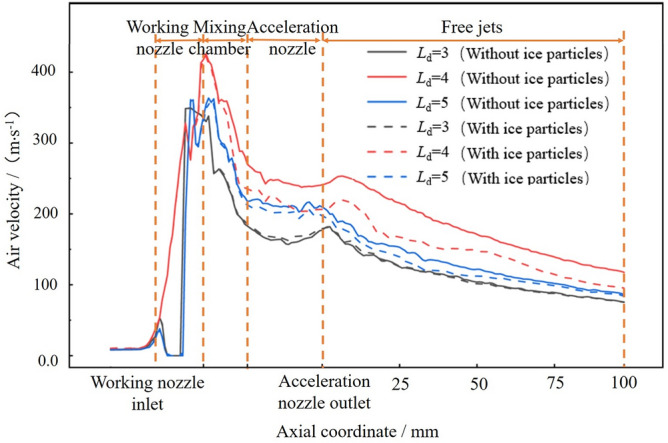
Axial velocity distribution of acceleration nozzle at different Ld.

**Figure 14:**
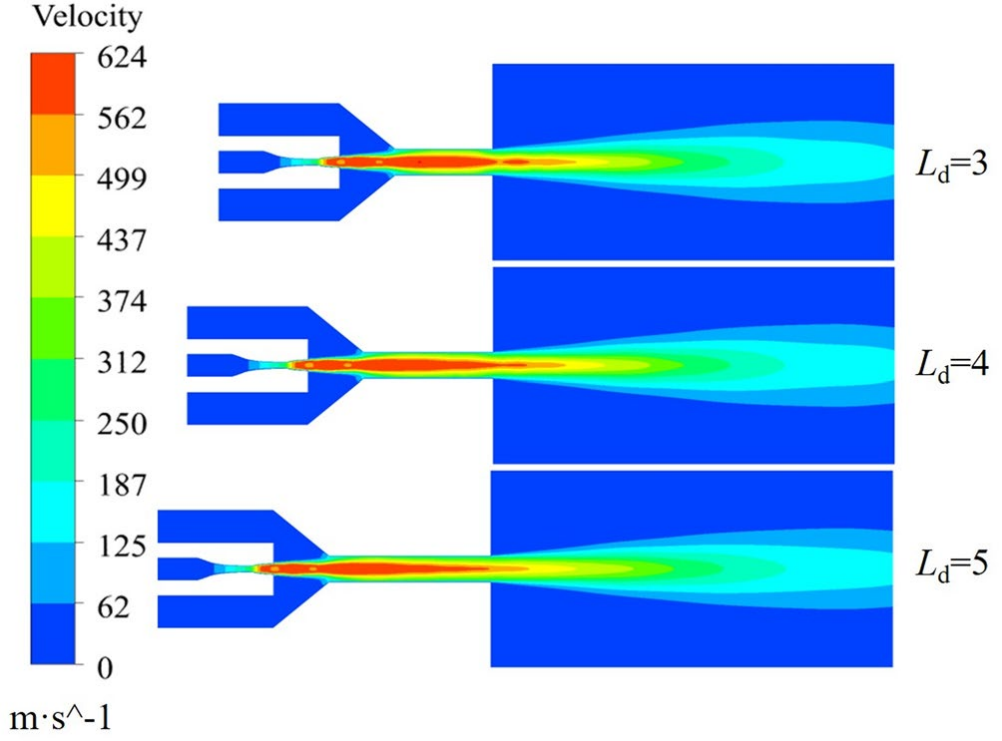
Airflow velocity clouds at different Ld.

The effect of the accelerating nozzle *L*_d_ ratio on the gas velocity is illustrated with *L*_n_ = 0 mm, *n* = 1.5 and *D*_n_ = 4.0, and the calculation results are shown in Fig. 13. The gas velocity starts to accelerate at the inlet of the working nozzle and reaches its maximum at the outlet of the working nozzle. After the supersonic gas enters the mixing chamber, the velocity begins to decrease gradually. It accelerates again after entering the accelerating nozzle, but the increase in velocity is not significant. In the free jet section, the gas velocity continues to decrease. The gas velocity is always highest at *L*_d_ = 4.0. As shown in the gas velocity cloud diagram at different *L*_d_ values in Fig. 14, at *L*_d_ = 4.0, the gas flow fluctuation is minimal, resulting in lower energy loss and a longer isovelocity core in the free jet stage. In contrast, at *L*_d_ = 3.0, the gas fluctuates significantly as it enters the free-flow field, resulting in lower jet energy. The gas velocity is always minimized at *L*_d_ = 5.0. Therefore, the gas flow field is more stable at *L*_d_ = 4.0.

##### Effects on ice particle acceleration

**Figure 15:**
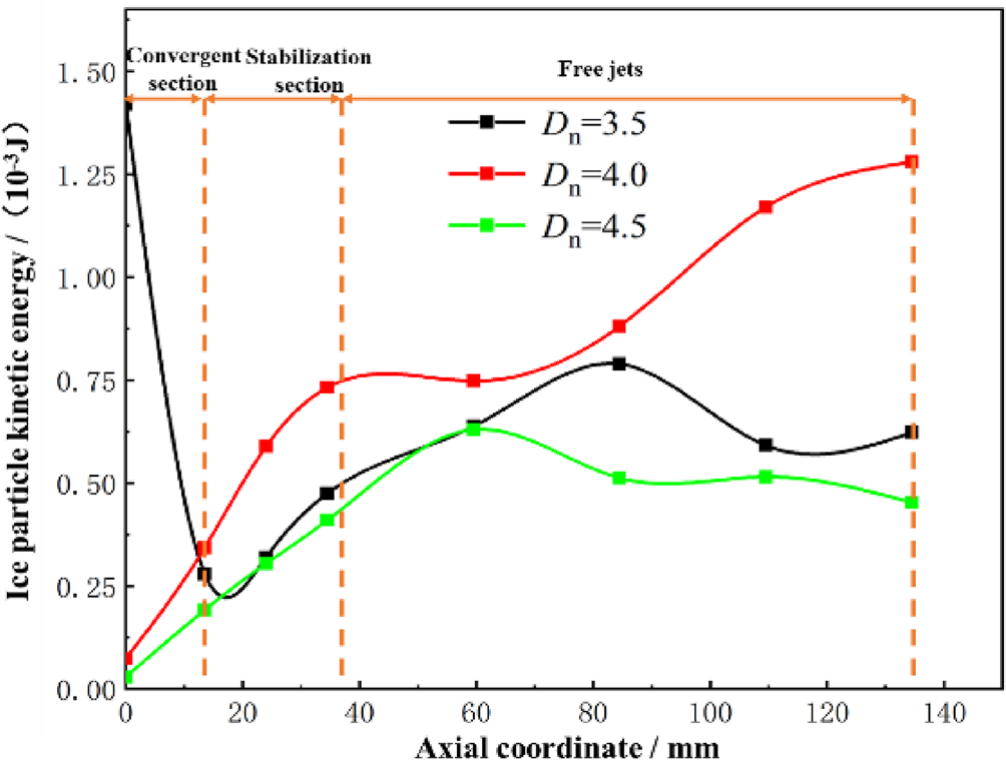
Variation of kinetic energy of ice particles with different Dn.

The effect of the accelerating nozzle diameter ratio on the kinetic energy of ice particle impact is illustrated with *L*_n_ = 0 mm, *n* = 1.5 and *L*_d_ = 4.0, and the calculated results are shown in Fig. 15. The kinetic energy of ice particles starts to increase after entering the accelerating nozzle, but decreases at *D*_n_ = 3.5. In the free jet stage, different *D*_n_ values have different effects on the kinetic energy of ice particles. The kinetic energy decreases at* D*_n_ = 3.5 and *D*_n_ = 4.5 at a larger target distance, but the velocity of ice particles consistently increases at *D*_n_ = 4.0, reaching a maximum impact kinetic energy of 1.28 × 10^-3^ J at a target distance of 100 mm. Combined with the analytical results in Section  2.5.1, it can be seen that different *D*_n_ values affect the spatial structure inside the accelerating nozzle, which in turn affects the airflow’s acceleration effect on the ice particles. At *D*_n_ = 4.0, the airflow fluctuation is minimal, and the kinetic energy of ice particles increases continuously, providing the best acceleration effect on ice particles.

**Figure 16:**
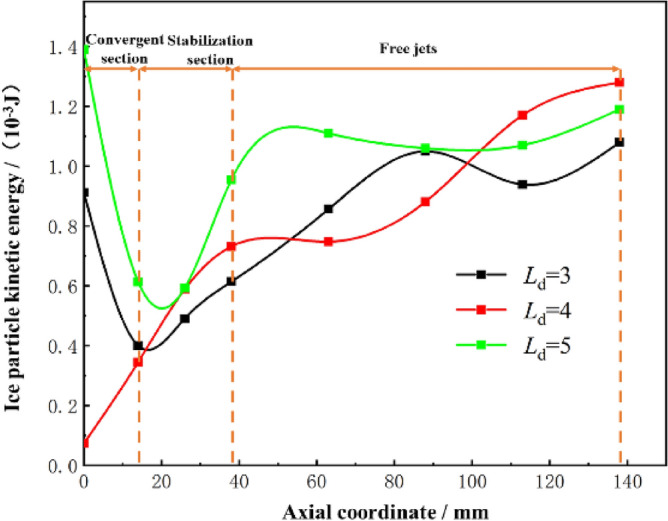
Variation of kinetic energy of ice particles with different Ld.

The effect of the length-to-diameter ratio of the accelerating nozzle on the kinetic energy of ice particle impact is illustrated with *L*_n_ = 0 mm, *n* = 1.5, *D*_n_ = 4.5 as an example, and the calculated results are shown in Fig. 16. After entering the accelerating nozzle, the kinetic energy of ice particles keeps increasing at *L*_d_ = 4.0, while the velocity of ice particles decreases and then increases at *L*_d_ = 3.0 and *L*_d_ = 5.0. In the free jet stage, the ice particle velocity changes significantly at different *L*_d_ values, and the ice particle kinetic energy fluctuates greatly at *L*_d_ = 3.0 and *L*_d_ = 5.0, but continues to increase at *L*_d_ = 4.0 reaching a maximum impact kinetic energy of 1.28 × 10^-3^ J at a target distance of 100 mm. Combined with the analytical results in Section  2.5.1, it can be seen that different accelerating nozzle *L*_d_ ratios affect the spatial morphology inside the accelerating nozzle and thus the acceleration of ice particles. At *L*_d_ = 4.0, the airflow fluctuation is minimal, achieving maximum impact kinetic energy, which provides the best effect on the acceleration of ice particles.

The results show that the optimal ejector structure is a working nozzle with *L*_d_ = 4.0, *D*_n_ = 4.0 and a pressure ratio of 1.5. At this configuration, the maximum impact kinetic energy achieved by the ice particles is 1.56 × 10^−3^ J.

## Ice pellet air jet paint stripping test

### Experimental system

**Figure 17:**
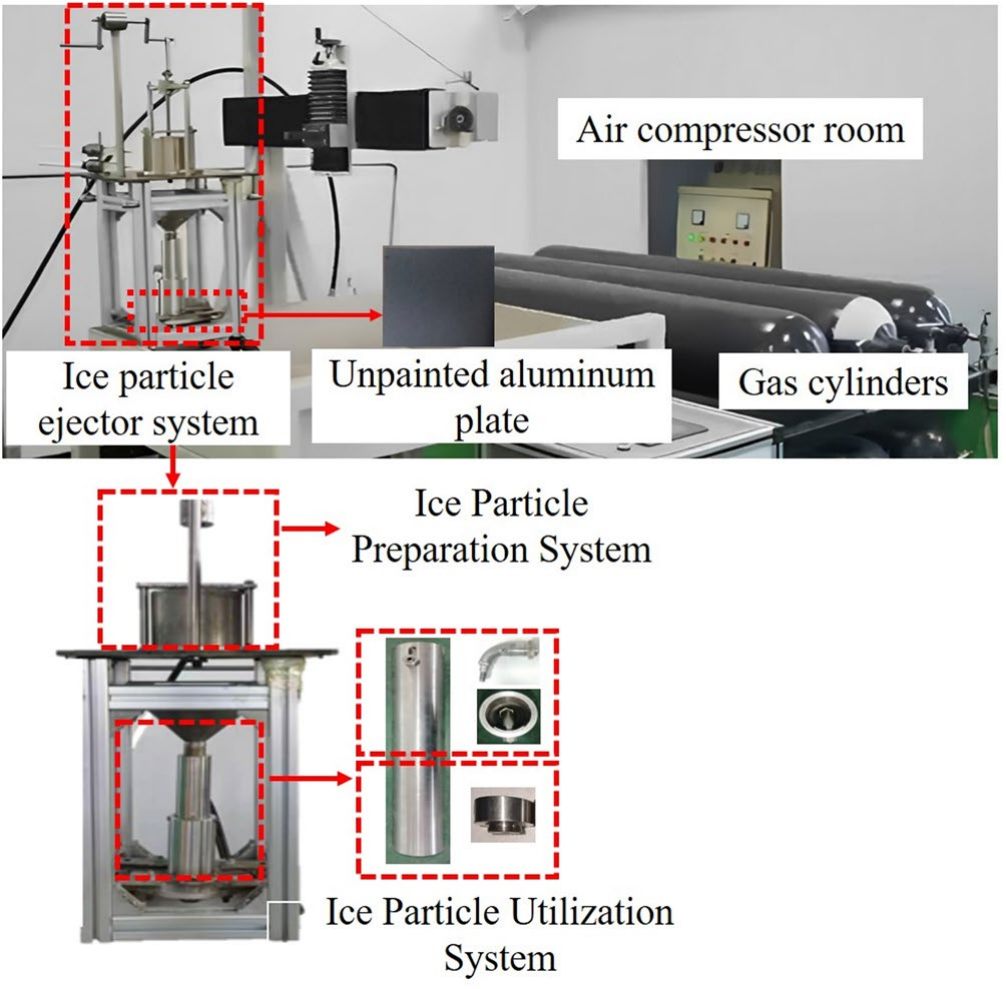
Ice Pellet Air Jet Paint Removal Test System.

The experimental system is shown in Fig. 17. It mainly consists of a gas supply system, an ice grain preparation system, an ice grain ejector system, and an experimental platform. The gas supply system provides compressed gas at a pressure of 2 MPa, regulated through a pressure-reducing valve. The test platform is used to fix the aluminum alloy specimen, which is a 10 cm × 10 cm aluminum alloy plate. Before the test, the aluminum alloy plate was uniformly sprayed with a paint thickness of 0.1 mm.

**Figure 18:**
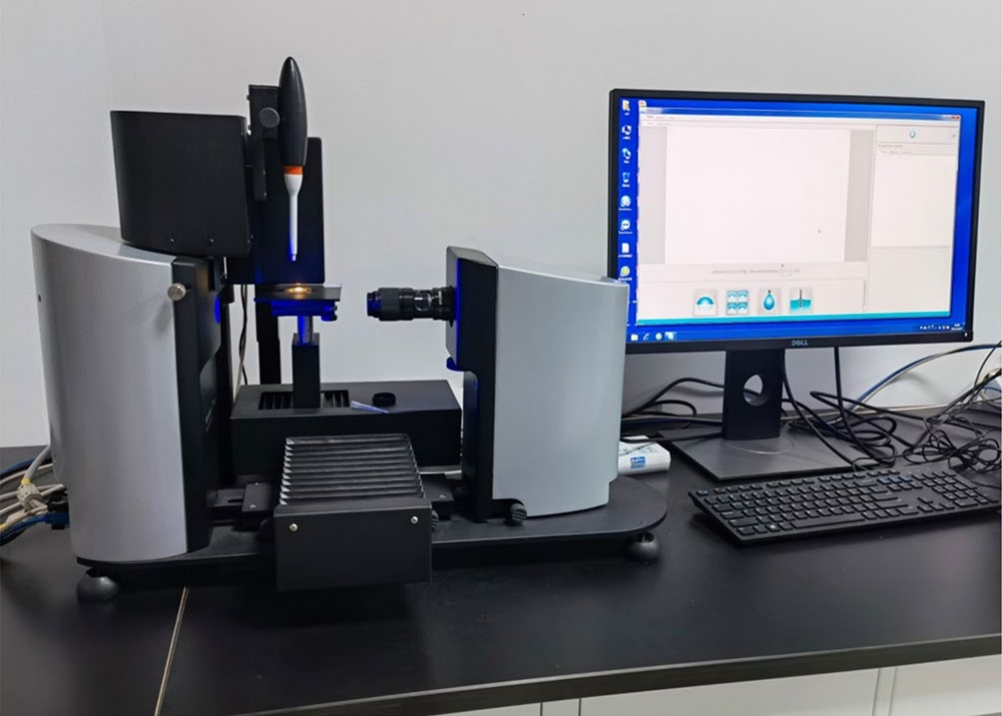
Test instrument for sample morphology.

To further analyze the effect of different ejector nozzle structures on the surface treatment of materials, the Attension Theta Optical Contact Angle Meter was used to test the roughness of the specimens treated with the ice-particle air jet. The device is shown in Fig. 18. The data from the measured area of the specimen were extracted, and three indicators were used to evaluate the surface treatment effect of the ice-particle air jet on aluminum alloys: *Ra*, which represents the arithmetic mean of the absolute value of the contour deviation within the sampling length; *Rp*, which represents the maximum peak of the contour, arithmetically, relative to the mean line within a sampling length; and *Sdr*, which represents the ratio of the interfacial area to the projected area.

### Experimental program

To obtain the optimal nozzle structure parameters for the ejector, an ice air jet paint removal test was conducted. The test program is shown in Table 3. The paint removal effect is characterized by the actual paint removal radius and the morphology and roughness of the metal surface after paint removal. A larger paint removal radius indicates a larger paint removal area per unit time, resulting in higher paint removal efficiency. Surface roughness is related to the impact kinetic energy of the ice particles; the smaller the surface roughness, the larger the impact kinetic energy of the ice particles, and the better the effect of ice air jet metal surface treatment. Because the spray paint thickness on the aluminum alloy surface was thin for this test and aimed mainly to verify the jet’s surface treatment ability, a small amount of damage to the surface material is considered normal.

**Table 3:** Experimental scheme.

Number	Inlet pressure/MPa	Standoffdistance /mm	Mass Flow/(g.s^-1^)	Impact time/s	*L*_n_/mm	*n*	*D*_n_	*L*_d_
1	2	100	10	10	− 5	1.5	4.5	4.0
2					0	1.0	4.0	5.0
3					5	0.8	3.5	3.0
4					− 5	0.8	3.5	3.0
5					0	1.0	4.5	4.0
6					5	1.5	4.0	5.0
7					− 5	0.8	3.5	3.0
8					0	1.5	4.0	4.0
9					5	1.0	4.5	5.0
10					− 5	0.8	3.5	3.0
11					0	1.5	4.0	4.0
12					5	1.0	4.5	5.0

### Test results and analysis

**Figure 19:**
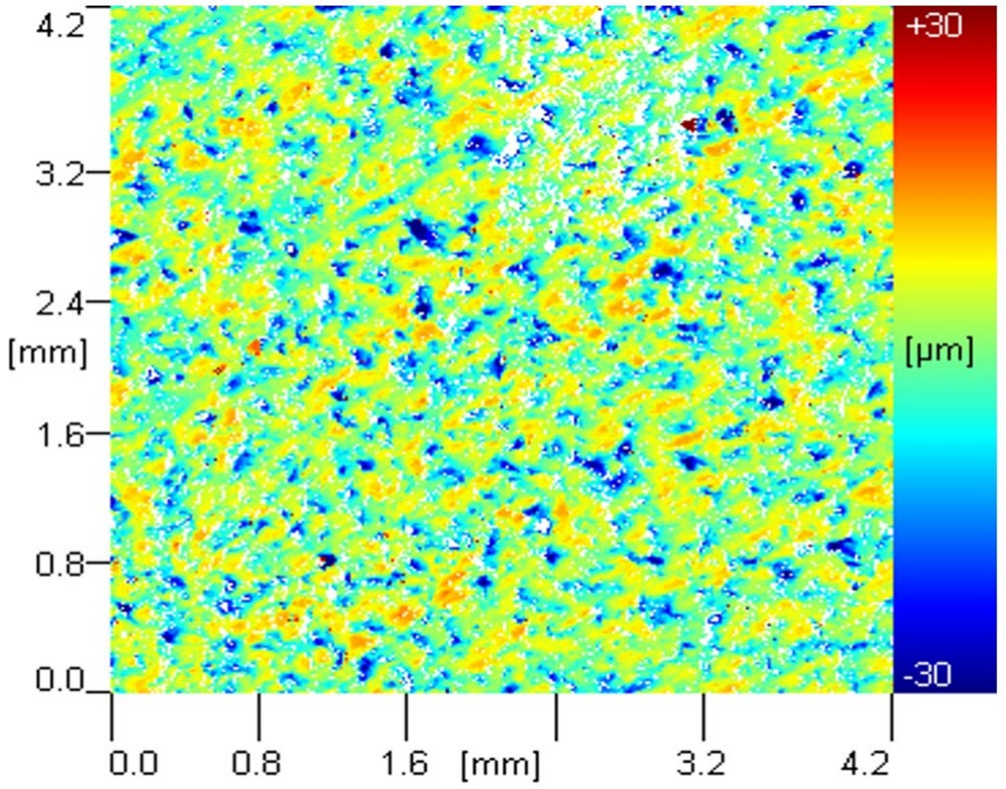
Morphology test results of aluminum alloy plate without erosion.

To compare the effects of the test, the morphology test results before erosion are shown in Fig. 19, and the roughness test results before erosion are shown in Table 4.

**Table 4:** Roughness test results of aluminum alloy plate without erosion.

Parameter	*Ra *(μm)	*Rp *(μm)	*Sdr*
Value	3.194 ± 0.489	4.327 ± 0.667	15.082 ± 2.173

#### ***Influence of L***_***n***_***、n on paint stripping effect***

**Figure 20:**
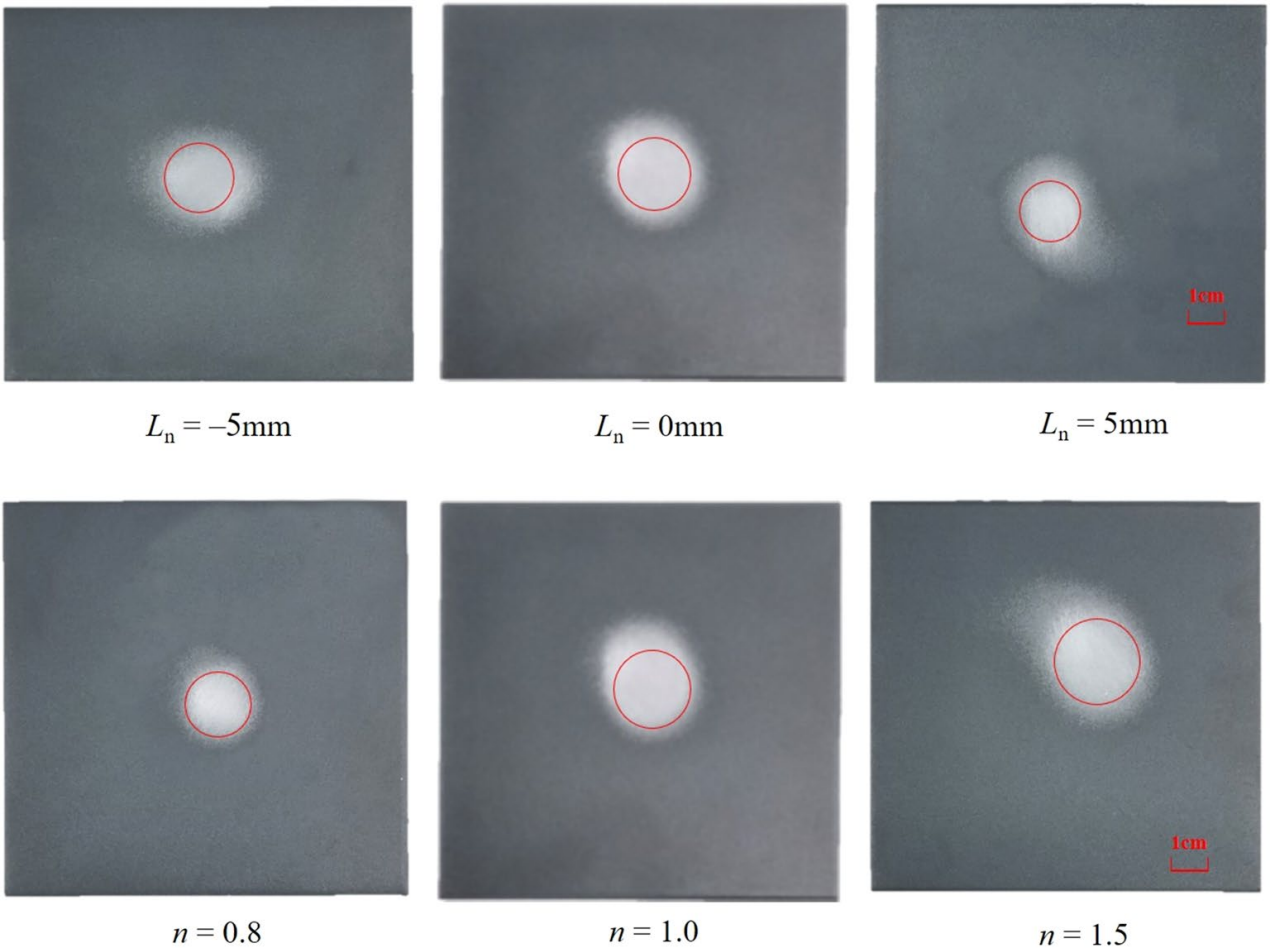
Paint removal effect under different Ln、n.

**Figure 21:**
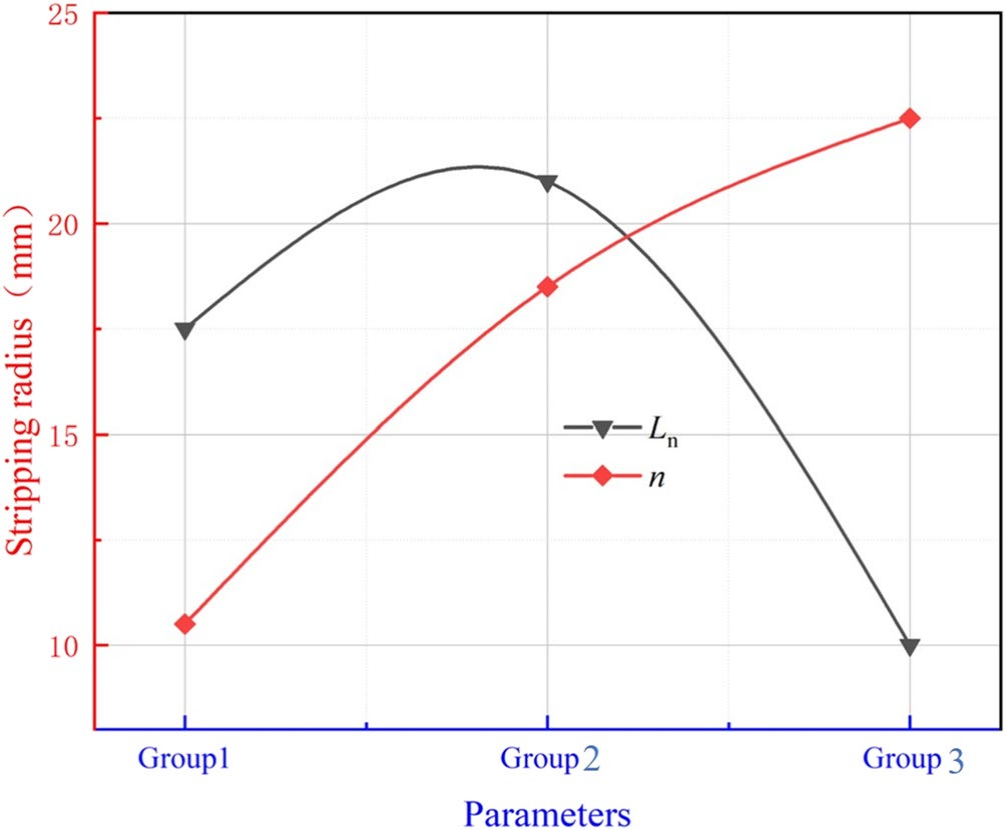
Paint removal radius under different Ln、n.

The paint removal effect on aluminum alloy specimens at different *L*_n_ and nnn values is shown in Fig. 20, and the data are presented in Fig. 21. The main purpose of this experiment is to verify that the designed jet structure can effectively remove paint. According to previous studies, the addition of obstacles has been used to improve the effective removal capability of the gas abrasive jet^38,39^. However, this requires specific conditions for the obstacles, such as their location and size not affecting the gas jet flow field, which is difficult to achieve in practice. Park et al.^40^ conducted experiments to describe the good performance of MAJM in glass micro-cutting grooves. Wakuda et al.^41,42^ performed MAJM on different engineering ceramics using three different abrasives to identify the material response of alumina ceramics to the impact of micro-abrasive grains and revealed the effect of workpiece properties on the machinability of engineering ceramics during the MAJM process. Compared to previous studies, this paper aims to verify that the designed structure provides better paint removal effects. The paint removal results achieved under the experimental conditions of this study are shown in Fig. 20. From Fig. 20, it can be seen that the radius of paint removal increases and then decreases with the increase of *L*_n_. The radius of the erosion area produced at *L*_n_ = 0 mm is the largest at 21 mm, and the erosion effect is also the best.

**Figure 22:**
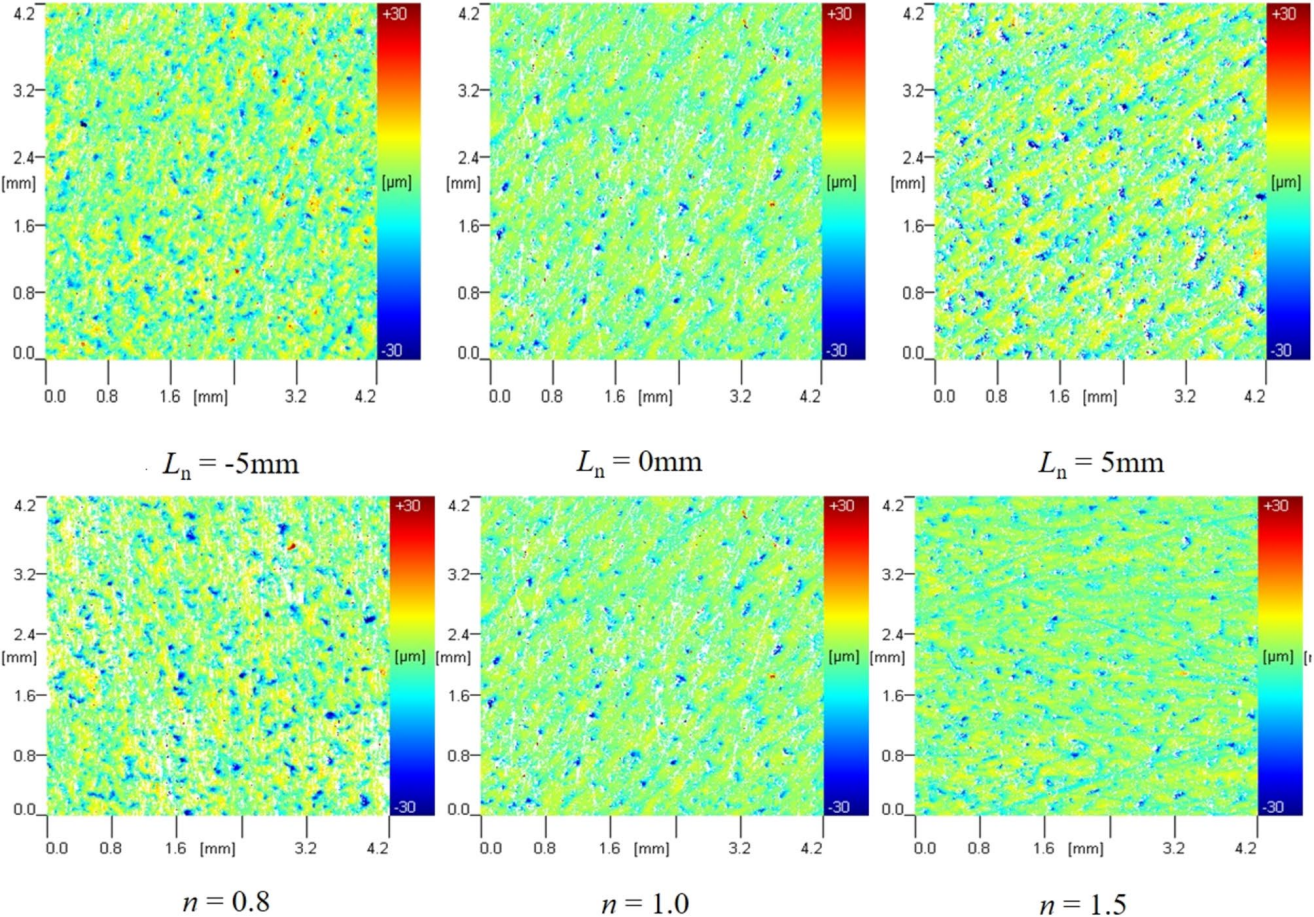
Morphological test results of aluminum alloy plate under different Ln、n.

Taking *L*_n_ = 0 mm, *L*_d_ = 4.0、*D*_n_** = **4.0 as examples to illustrate the effect of *n* on the paint removal effect, the paint removal effect of the ice air jet under different *n* values increases with the increase of *n*. The surface treatment effect of the ice air jet is best when the pressure ratio *n* = 1.5, resulting in more thorough paint removal in the eroded area. The surface morphology and roughness of the specimens were tested, with the results of the surface morphology test shown in Fig. 22 and the results of the roughness test shown in Table 5.

**Table 5:** Roughness test results of aluminum alloy plate under different working nozzle structures.

Parameter	Value	*Ra *(μm)	*Rp *(μm)	*Sdr*
*L*_n_(mm)	− 5	1.649 ± 0.383	2.892 ± 0.525	7.846 ± 1.457
	0	1.437 ± 0.365	2.458 ± 0.536	6.484 ± 1.327
	5	1.683 ± 0.427	2.935 ± 0.615	8.942 ± 1.812
*n*	0.8	1.742 ± 0.461	3.046 ± 0.497	8.957 ± 1.611
	1.0	1.437 ± 0.365	2.458 ± 0.536	6.484 ± 1.327
	1.5	1.156 ± 0.136	1.782 ± 0.561	5.652 ± 1.132

From the test results, it can be seen that the roughness of the aluminum alloy plate decreases and then increases as *L*_n_ = 0 mm increases, with the surface roughness being the minimum at *L*_n_ = 0 mm, measured at 1.437 ± 0.365 μm. From the morphology and roughness test results of the aluminum alloy specimens at different nnn values, it can be seen that the surface roughness decreases consistently with the increase of *n*. At *n* = 1.5, the surface roughness is minimized at 1.156 ± 0.136 μm.

#### Influence of Dn、Ld on paint stripping effect

**Figure 23:**
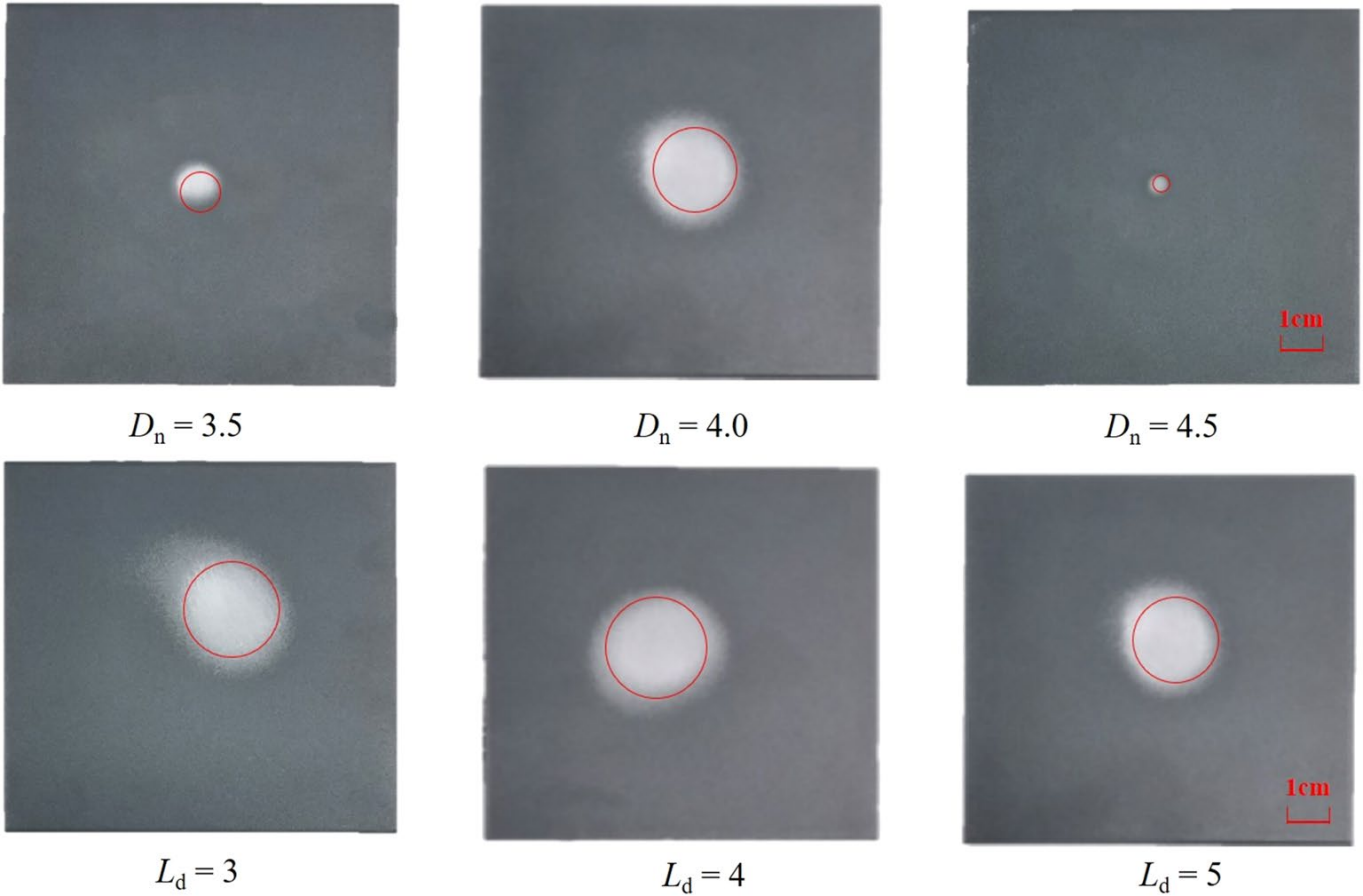
Paint removal effect with different Dn、Ld.

**Figure 24:**
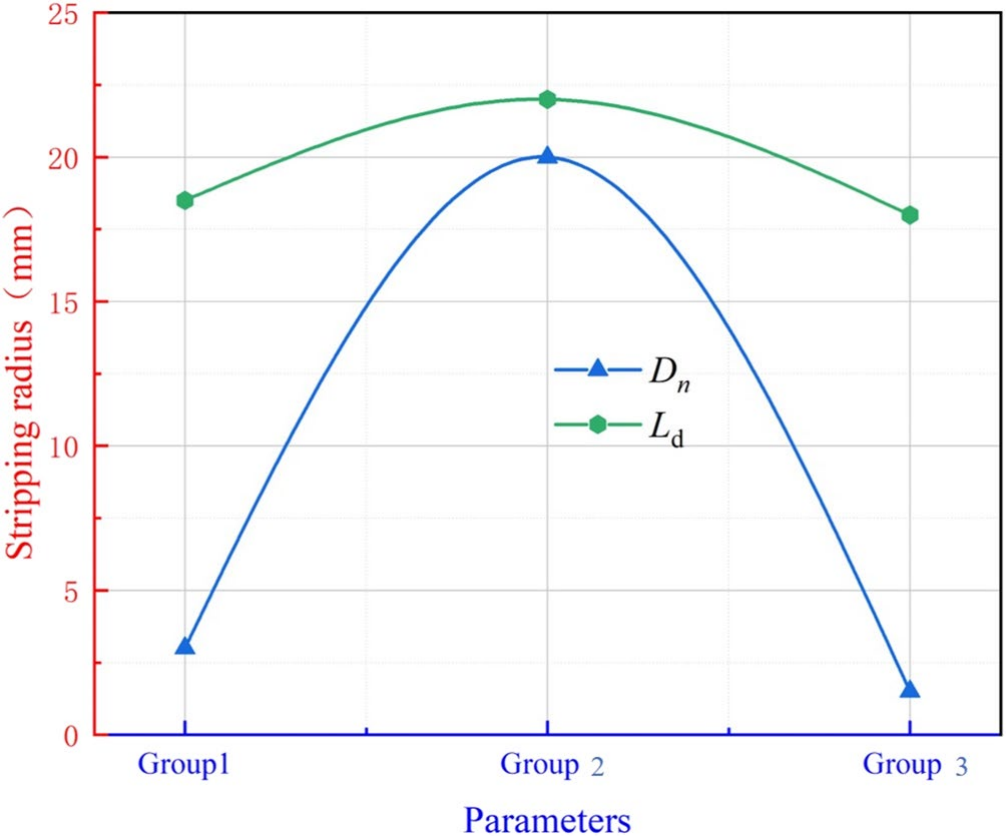
Paint removal radius with different Dn、Ld.

The paint removal effect of the specimens under different *D*_n_ and* L*_d_ values is shown in Figs. 23 and 24. From Fig. 24, it can be seen that the radius of paint stripping increases and then decreases with the increase of *D*_n_, with the maximum radius of paint stripping being 20 mm when *D*_n_ = 4.0.

Taking *L*_n_ = 0 mm, *D*_n_ = 4.0 and n = 1.5 as examples to illustrate the effect of *L*_d_ on the paint removal effect, Fig. 24 shows that at a given target distance, the paint stripping radius first increases and then stabilizes with the increase of *L*_d_, When the accelerated nozzle aspect ratio *L*_d_ = 4.0 , the paint stripping radius can reach 23 mm.

**Figure 25:**
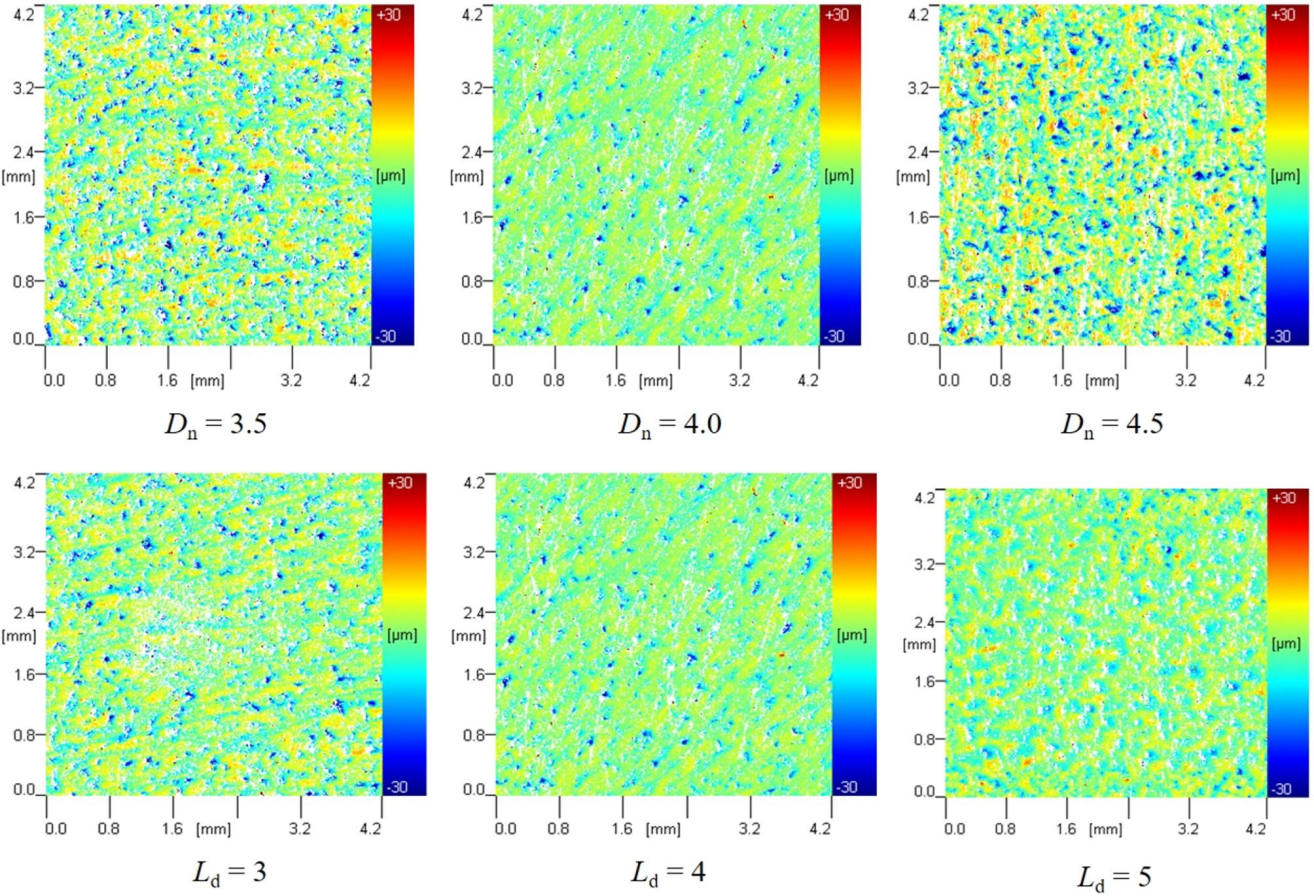
Morphological test results of aluminum alloy plate under different Dn、Ld.

The specimens were then tested for surface morphology and roughness. The results of the surface morphology test are shown in Fig. 25, and the results of the roughness test are shown in Table 6. It can be seen that as *D*_n_ increases, the roughness of the aluminum alloy plate decreases and then increases, with the surface roughness being the smallest at *D*_n_ = 4.0 mm, measured at 1.437 ± 0.365 μm; As *L*_d_ increases, the roughness of the aluminum alloy plate first decreases and then increases, with the surface roughness being the minimum at *L*_d_ = 4.0, measured at 1.437 ± 0.365 μm.

**Table 6:** Results of aluminum alloy plate roughness test under different accelerating nozzle structures.

Parameters	Value	*Ra *(μm)	*Rp *(μm)	*Sdr*
*D*_n_	3.5	2.134 ± 0.426	3.516 ± 0.621	10.251 ± 1.632
	4.0	1.437 ± 0.365	2.458 ± 0.536	6.484 ± 1.327
	4.5	2.584 ± 0.495	3.861 ± 0.579	11.325 ± 2.782
*L*_d_	3.0	1.525 ± 0.176	2.657 ± 0.543	7.484 ± 1.534
	4.0	1.437 ± 0.365	2.458 ± 0.536	6.484 ± 1.327
	5.0	1.461 ± 0.316	2.462 ± 0.541	6.831 ± 1.342

## Conclusion

- (1) An instantly prepared and instantly utilized ice pellet jet surface treatment technology is proposed to solve the problems of ice pellet bonding and clogging in traditional ice pellet jet paint removal technology.
- (2) Numerical simulation studies were carried out using a coupled Fluent-EDEM method to derive the optimal ejector structure parameters that can sufficiently eject accelerated ice particles at 2 MPa.
- (3) Through the aluminum alloy paint stripping test, it was verified that the designed ejector structure can change the surface roughness of the aluminum alloy plate from 3.194 ± 0.489 μm to 1.156 ± 0.136 μm, demonstrating a superior surface treatment effect.
- (4) Jet structures for instant preparation and utilization of ice particles can provide theoretical and technological support in the field of ice particle air jet surface treatment.

## Data Availability

All data included in this study are available upon request from the corresponding author.
